# Aging of a Bacterial Colony Enforces the Evolvement of Nondifferentiating Mutants

**DOI:** 10.1128/mBio.01414-19

**Published:** 2019-09-03

**Authors:** Rachel Hashuel, Sigal Ben-Yehuda

**Affiliations:** a Department of Microbiology and Molecular Genetics, Institute for Medical Research Israel-Canada, The Hebrew University-Hadassah Medical School, The Hebrew University of Jerusalem, Jerusalem, Israel; University of Washington

**Keywords:** *Bacillus subtilis*, colony, long-term stationary phase, sporulation, *spo0A*

## Abstract

Bacteria in nature are known to survive for long periods under restricting conditions, mainly by reducing their growth rate and metabolic activity. Here, we uncover a novel strategy utilized by bacterial cells to resist aging by propagating rather than halting division. Bacterial aging was monitored by inspecting colonies of the Gram-positive soil bacterium Bacillus subtilis, which is capable of differentiating into various cell types under nutrient exhaustion. We revealed that after days of incubation, rejuvenating subpopulations, arrayed over the mother colony, emerged. These subpopulations were found to harbor mutations in a variety of genes, restricting the ability of the cells to differentiate. Surprisingly, even mutations that are not classically designated to developmental pathways, concluded in differentiation deficiency, indicating that multiple paths can reach this same outcome. We provide evidence that the evolved mutants continue to divide under conditions that favor entry into quiescence, hence becoming abundant within the aging population. The occurrence of such nondifferentiating mutants could impact bacterial population dynamics in natural niches.

## Abstract (precis)

Until now, bacterial cells facing nutrient deprivation were shown to enter dormancy as a strategy to survive prolonged stress, with the most established examples being sporulation, stationary phase, and persistence. Here, we uncovered an opposing strategy for long-term bacterial survival, in which mutant subpopulations cope with a challenging niche by proliferating rather than by stalling division. We show that this feature stems from mutations in genes disturbing the capability of the cells to differentiate into a quiescent state, enabling them to divide under restrictive conditions. Our study challenges the dogma of bacterial aging by highlighting an additional survival strategy resembling that of cancerous cells in animal organs.

## INTRODUCTION

Bacteria constantly compete for vital nutrients and yet can persist for long periods once nutrients have been exhausted (1). Various approaches were developed by bacteria to cope with challenging surroundings, with the most common one being residing in stationary phase, characterized by decreased metabolic activity and increased resistance to various stresses (2–4). After prolonged exposure to starvation, bacteria can enter into long-term stationary phase, which is typically associated with global modulation of gene expression and metabolic activity (5–9). Furthermore, phenotypes such as growth arrest, increased population heterogeneity, tolerance to antibiotics and oxidative stress, as well as an elevated mutation rate, were attributed to this phase (10–15). In some bacteria, mainly in Escherichia coli, phenotypic changes during this phase were found to be associated with genetic mutations, conferring a growth advantage in stationary phase phenotype (GASP) (16–19). Only a few genes have been implicated in the GASP phenotype, including mutations located in genes involved in metabolic pathways, as well as in the stress response transcriptional regulator *rpoS*, and yet the growth advantage due to *rpoS* alterations is not fully understood (18, 20). A more global analysis of mutations acquired to enable rejuvenation of aging populations is missing.

Upon starvation, the Gram-positive soil bacterium Bacillus subtilis can differentiate into various forms corresponding to stationary phase, competence, or biofilm formation (21–25). Moreover, B. subtilis and its relatives developed an alternative strategy to resist nutrient deprivation by forming a metabolically dormant cell type known as a spore, allowing the organism to endure harsh environments and to revive when conditions become favorable (26, 27). However, the spore rarely acquires new features or undergoes genetic alterations, whereas bacterial cells facing stress can adapt to new conditions and evolve. Intriguingly, all the alternative cellular differentiation pathways in B. subtilis are under the regulation of the same transition state regulator Spo0A. The activity of Spo0A is controlled by a phosphorelay, consisting of the major sporulation kinase KinA that autophosphorylates and transfers the phosphoryl group to Spo0A via the intermediate proteins Spo0F and Spo0B. Each alternative cellular fate is determined by the different phosphorylation level of this transcriptional regulator (28–31). The perseverance of various strategies to cope with nutrient limitation suggests that coexistence of a variety of differentiated cell types is needed for long-term species durability.

Here, we examined the aging process of cells within B. subtilis colonies, a preferential form of multicellular community, enabling the accommodation of phenotypic and genetic diversity and the independent emergence of mutant clones (see, for example, references 32, 33, 34, 35, 36, and 37). We revealed the appearance of redividing subpopulations harboring mutations in an array of genes, crucial for cellular differentiation, including sporulation, competence, and/or stationary phase. These mutants were found to adopt features, allowing their propagation despite nutrient shortage. We propose that the emergence of rejuvenating populations, appearing during colony aging, emanates from mutants locked in a nondifferentiating state.

## RESULTS

### Aging bacterial colonies give rise to rejuvenating microcolonies.

**FIG 1:**
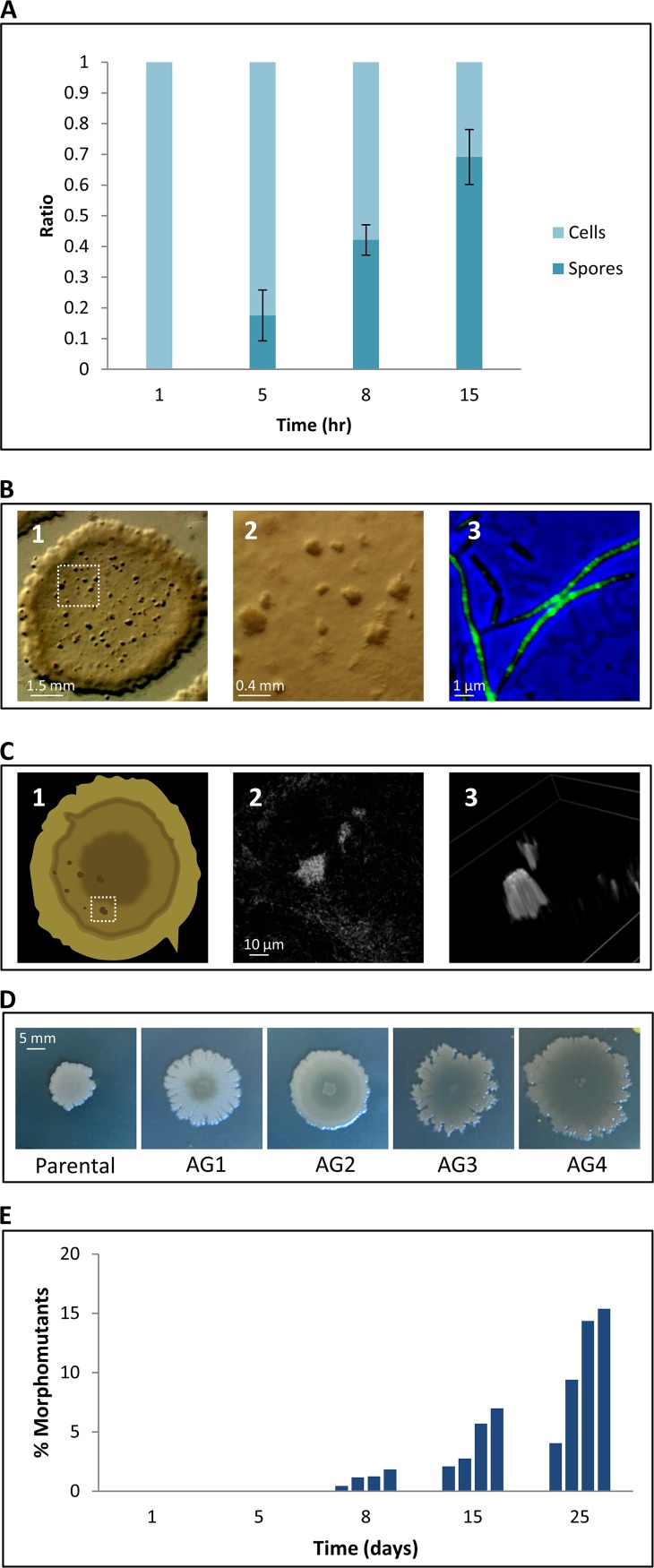
Investigating the features of aging colonies. (A) Cells of aging WT (PY79) colonies were collected at the indicated days, and samples were monitored using light microscopy. The ratios between cells and bright spores are shown. The results are average values and the SD of three independent experiments. (B) Colonies of the NS FtsZ-GFP-producing strain (RU9) were incubated for 25 days and visualized over time using a stereomicroscope and fluorescence microscopy. An image of a typical 25-day-old single colony (panel 1), a magnification of the inset box from panel 1 highlighting the formation of microcolonies (panel 2), and an overlay image of phase-contrast (blue) and FtsZ-GFP (green) of cells collected from 25-day-old colonies (panel 3) are shown. (C) Fifteen-day-old colonies of the NS FtsZ-GFP producing strain (RU9) were subjected as a whole to two-photon microscopy analysis. Schematics of a colony with a typical region (white square) visualized by the two-photon microscopy (panel 1) and the foci of cells expressing FtsZ-GFP within the depth of a colony from two different angles (panels 2 and 3) are shown. (D) Images of NS (RU9) strain and four representative morphomutant colonies, named AG1-4 (Fig. 2A and Table S1B), forming unique shape variations after 8 days of incubation. (E) Colonies of NS (RU9) were replated at the indicated days, and the ratio of morphomutant colonies in the entire progeny colonies population was calculated. Each bar represents the ratio of morphomutants derived from an aging colony at the indicated day. Four colonies were examined each day.

To initially characterize community features of the aging bacterial colony, we investigated the ratio between spores and nonsporulating cells during the course of 15 days. As expected, the number of spores increased over time; however, even after 15 days of incubation, when nutrients became limited, approximately 30% of the colony inhabitants were nonsporulating cells (Fig. 1A

10.1128/mBio.01414-19.10 TABLE S1 Bacterial strains and primers used in this study. Download Table S1, DOCX file, 0.1 MB. Copyright © 2019 Hashuel and Ben-Yehuda. 2019 Hashuel and Ben-Yehuda This content is distributed under the terms of the Creative Commons Attribution 4.0 International license.;

see also Fig. S1A to C in the supplemental material). This is consistent with the mixture of spores and dividing cells previously observed in developing B. subtilis colonies that were prompted to sporulate (38). To isolate exclusively the nonsporulating subpopulation, we used a sporulating but nongerminating (NG; RU124) strain, from which only cells that did not enter sporulation could be recovered. Further, assuming that spore germination continuously occurs during aging, such strain reports the total accumulation of spores throughout the colony life span. Interestingly, a portion of nonsporulating cells similar to that of the wild type (WT) was monitored in this strain at all tested time points (Fig. S1D), indicating that germination has a low impact on colony composition. We hypothesized that these nonsporulating cells provide the reservoir needed to enable the population to evolve under harsh conditions. To characterize the nonsporulating population, we followed aging in colonies derived from a nonsporulating mutant strain (NS; RU9), which is able to properly enter into sporulation but stalls at later stages (Fig. S1C and E) (27, 39). Cell division was tracked utilizing a green fluorescent protein (GFP) fusion to the major cell division protein FtsZ (40), and the colony architecture, as well as the morphology of individual cells, was monitored over time. Intriguingly, we visualized the emergence of small microcolonies arrayed over the mother colony, starting at approximately day 15, a phenomenon that was manifested as the colony aged (Fig. 1B, subpanels 1 and 2, and Fig. S2A and B). Concomitantly, along with the accumulation of lysed cells, we observed the frequent reappearance of elongated dividing cell chains harboring FtsZ rings (Fig. 1B, subpanel 3, and Fig. S2C). Two-photon microscopy detected foci of FtsZ-GFP-expressing cells within the depth of the old colony, signifying centers of highly dividing cells that correspond to the emerging microcolonies (Fig. 1C). As a comparison, an evenly distributed FtsZ-GFP signal was observed in a day-old colony (Fig. S2D). Importantly, the formation of microcolonies was also evident in aging colonies derived from NG and WT strains (Fig. S2E). Taken together, these results show that in the course of colony aging, subpopulations regain the ability to divide, leading to the formation of visible microcolonies.

10.1128/mBio.01414-19.1 FIG S1 Fraction of sporulating and nonsporulating cell populations within aging colonies. (A) Cells of aging colonies of WT (PY79), NS (RU9), and sporulating but nongerminating (NG, RU124) strains were collected at the indicated days, and similar OD_600_ values were plated for CFU count. The results are average values and SD from three independent experiments. (B and C) Cells of aging colonies of (B) NG (RU124) and (C) NS (RU9) strains were collected at the indicated days. Cells were heat treated (heat kill, HK) or untreated (No HK) and plated for CFU count. The results are average values and SD from three independent experiments. (D) Cells of aging WT (PY79) and NG parental (RU124) colonies were collected at the indicated days, and samples were followed by light microscopy. Shown is the ratio of bright spores in the entire cell population. The results are average values and the SD of three independent experiments. (E) Cells of aging colonies of WT (SB306) and NS (RU125), harboring P*_racA_-gfp*, were collected at the indicated days and visualized by light microscopy. RacA expression is induced upon entry to sporulation prior to polar septum formation (S. Ben-Yehuda, DZ. Rudner, and R. Losick, Science **299**:532–536, 2003, doi: 10.1126/science.1079914). Shown are overlay images of phase-contrast (blue) and GFP (green) of cells collected from aging colonies. Of note, the GFP signal indicates proper entry into sporulation in WT (SB306) and in NS (RU125) aging colonies, although no mature spores are eventually formed in the latter strain. Download FIG S1, TIF file, 2.5 MB. Copyright © 2019 Hashuel and Ben-Yehuda. 2019 Hashuel and Ben-Yehuda This content is distributed under the terms of the Creative Commons Attribution 4.0 International license.

10.1128/mBio.01414-19.2 FIG S2 Old colonies display microcolonies that correlate with the appearance of rejuvenating cells. (A) Fifteen-day-old NS (RU9) colonies were incubated and visualized by using a stereomicroscope at the indicated days. Shown are images of a single typical colony throughout time. (B) Magnification of the inset in panel A (15 days), highlighting the formation of microcolonies. Arrows indicate apparent microcolonies. (C) Colonies of NS (RU9) strain harboring *ftsZ-gfp* were incubated for 15 days. Cells were harvested and visualized by light microscopy at the indicated days. Shown are overlay images of phase-contrast (blue) and signals from FtsZ-GFP (green). Of note, accumulation of lysed cells is prominent from day 5 and forwards. (D) One-day-old colonies of the NS FtsZ-GFP-producing strain (RU9) were subjected as a whole to two-photon microscopy analysis. Shown are schematics of a colony with a typical region (highlighted with white square) visualized by two-photon microscopy (panel 1), and cells expressing FtsZ-GFP within the depth of a colony from two different angles (panels 2 and 3). The GFP signal seems to be evenly spread in the colony. (E) Fifteen-day-old colonies of WT (PY79) (panel 1) and NG (RU124) parental (panel 2) strains were visualized by a stereomicroscope. Shown are segments of typical 15-day-old colonies displaying apparent microcolonies. Microcolonies are highlighted by arrows. Download FIG S2, TIF file, 2.4 MB. Copyright © 2019 Hashuel and Ben-Yehuda. 2019 Hashuel and Ben-Yehuda This content is distributed under the terms of the Creative Commons Attribution 4.0 International license.

### Emergence of morphomutants from aging colonies.

The appearance of actively dividing microcolonies suggests that adaptive populations, evolved within the aging colony, could be the source of this occurrence. To enrich and select for the aging colony survivors, we replated 25-day-old colonies from the NS parental strain harboring multiple microcolonies. The analysis uncovered the emergence of progeny colonies, displaying morphologies different from the WT (Fig. 1D and Fig. S3A). Further examination showed that these atypical colonies accumulated over time, reaching 5 to 20% of the population at day 25 (Fig. 1E and Fig. S3B and C). These altered colonies were mostly associated with microcolony-enriched regions (Fig. S3B and C), suggesting that they originated from the rejuvenating cells. The appearance of such a morphologically distinct population was also evident in aging colonies stemming from the WT and from NG parental strains (Fig. S3A). Examination of the modified colonies revealed that the morphology was already detected on the first day of plating, indicating the existence of genetic mutations. We therefore termed the mutants yielding these atypical colonies “morphomutants.”

10.1128/mBio.01414-19.3 FIG S3 Morphomutants emerge from aging colonies. (A) Twenty-five-day-old colonies of NS (RU9), NG (RU124), and WT (PY79) parental strains were suspended and replated to form progeny colonies. Some of the progeny colonies displayed altered morphologies (morphomutants). Arrows indicate morphomutants. (B) Representation of the procedure used to estimate the ratio of morphomutant colonies. Twenty-five-day-old colonies of NS (RU9) were replated, and the ratio of morphomutant colonies from the entire progeny colonies population was calculated (Whole). For additional 25-day-old parental colonies, regions enriched in microcolonies (micro^+^) and regions without notable microcolonies (micro^–^) of the same colony were separately replated, and morphomutant ratio was calculated. (C) Results of the analysis described in panel B. The graph shows the median and 95% CI of ratios from 16 whole colonies and 9 colonies separated to micro^+^ and micro^–^ regions. Significance was evaluated by a two-tailed Mann-Whitney test, *P* = 0.00145. *n* ≥ 200 progeny colonies counted for each mother colony. Each colony in micro^+^ and micro^–^ is indicated with the same color code. (D) Upper panels show images of NS (RU9) strain and four representative morphomutants colonies, forming unique shape variations after 8 days of incubation (identical to Fig. 1D). Lower panels show images of WT (PY79) strain and four corresponding morphomutants, reconstructed by site-directed mutagenesis in the WT background, that recapitulate the typical morphomutant colony morphology after 8 days of incubation. Download FIG S3, TIF file, 3.0 MB. Copyright © 2019 Hashuel and Ben-Yehuda. 2019 Hashuel and Ben-Yehuda This content is distributed under the terms of the Creative Commons Attribution 4.0 International license.

### Identification of genetic mutations acquired by the morphomutants.

**FIG 2:**
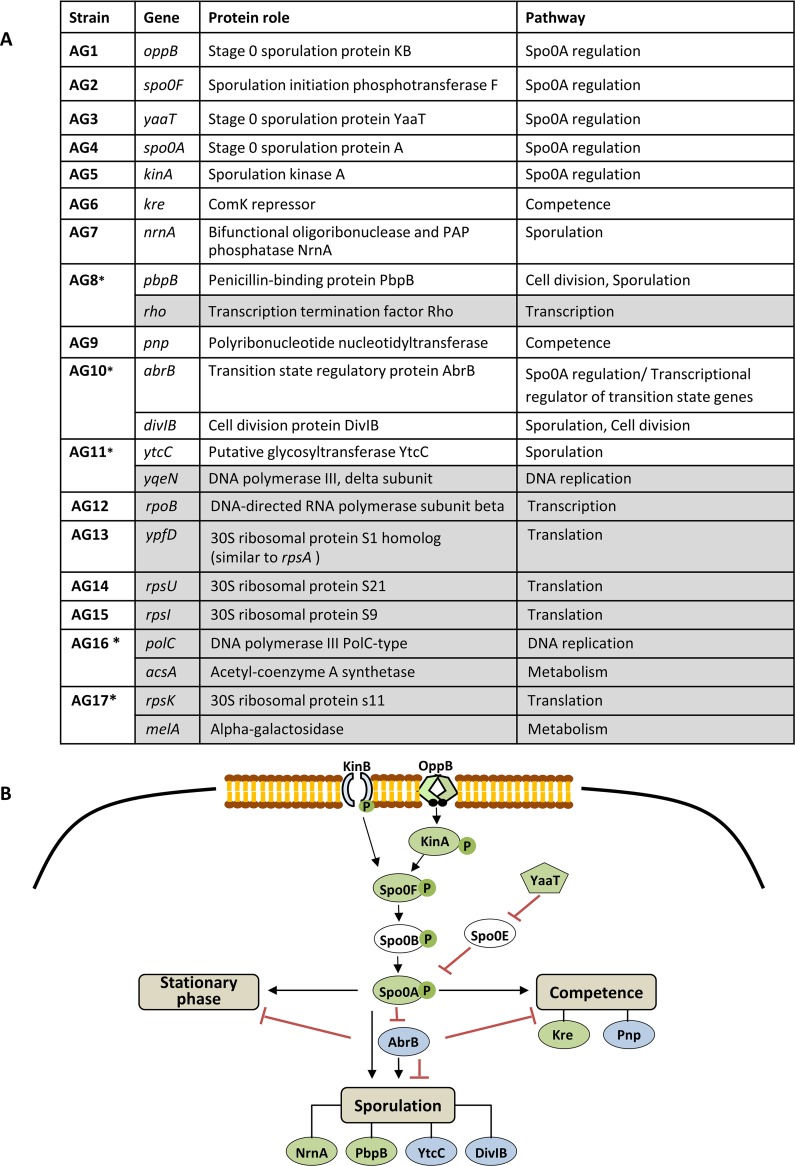
Genotypes of aging-induced morphomutants. (A) Seventeen morphomutants were isolated: 12 from an NS (RU9) background (AG1 to AG8 and AG12 to AG15) and 5 from an NG (RU124) background (AG9 to AG11, AG16, and AG17) and subjected to WGS analysis. A list of mutated genes found to participate in the Spo0A differentiation pathway (white background) and in basic cellular processes of replication, transcription, translation, and metabolism (gray background) is presented. *Strains that harbor two mutations. Gene names, protein roles, and pathways are based on UniProt and SubtiWiki database information. (B) Of 22 morphomutations, 12, located in 11 strains, were assigned to genes encoding proteins that participate in the Spo0A differentiation pathway. A representation of the Spo0A phosphorelay and its downstream differentiation pathways is shown. Factors in which mutations were found in their corresponding genes are indicated in green (NS background) or blue (NG background).

The identified morphomutants are likely to harbor selective advantage mutations, enabling them to propagate under nutrient deprivation. To uncover the mutated genes, whole-genome sequence (WGS) analysis was carried out on 17 independent isolates derived from the NS (RU9) parental strain, as well as from the NG (RU124) strain, shown to have a spore/nonsporulating cell ratio similar to that of the WT (Fig. S1D), and as such reflects the natural composition of aging colonies. The analysis revealed single or double mutations within the genome of each of the morphomutants, assigned to different genes (Fig. 2A and Table S1B). Remarkably, further investigation enabled us to sort the majority (12/22 located in 11 strains) of the mutated genes to be part of the Spo0A differentiation pathway (Fig. 2). The remaining mutations (10/22 located in eight strains) were situated in genes required for basic cellular processes of replication, transcription, translation, and metabolism (Fig. 2A and Table S1B).

The identified mutations belonging to the Spo0A differentiation pathway were located in genes encoding the major Spo0A phosphorelay components (e.g., *spo0A*, *kinA*, and *spo0F*), as well as in the central transition state regulator *abrB*, and in downstream components required for various Spo0A regulated developmental transitions (e.g., *pnp* and *pbpB*) (Fig. 2B). Reconstruction of several of the obtained alleles (i.e., *oppB*, *spo0F*, *yaaT*, and *spo0A*) in a WT background recapitulated the morphomutant colony morphology, indicating that these alleles cause this distinctive phenotype (Fig. S3D). These results show that morphomutants, deficient in the Spo0A pathway, frequently evolve in aging colonies.

### Morphomutants are deficient in differentiation.

**FIG 3:**
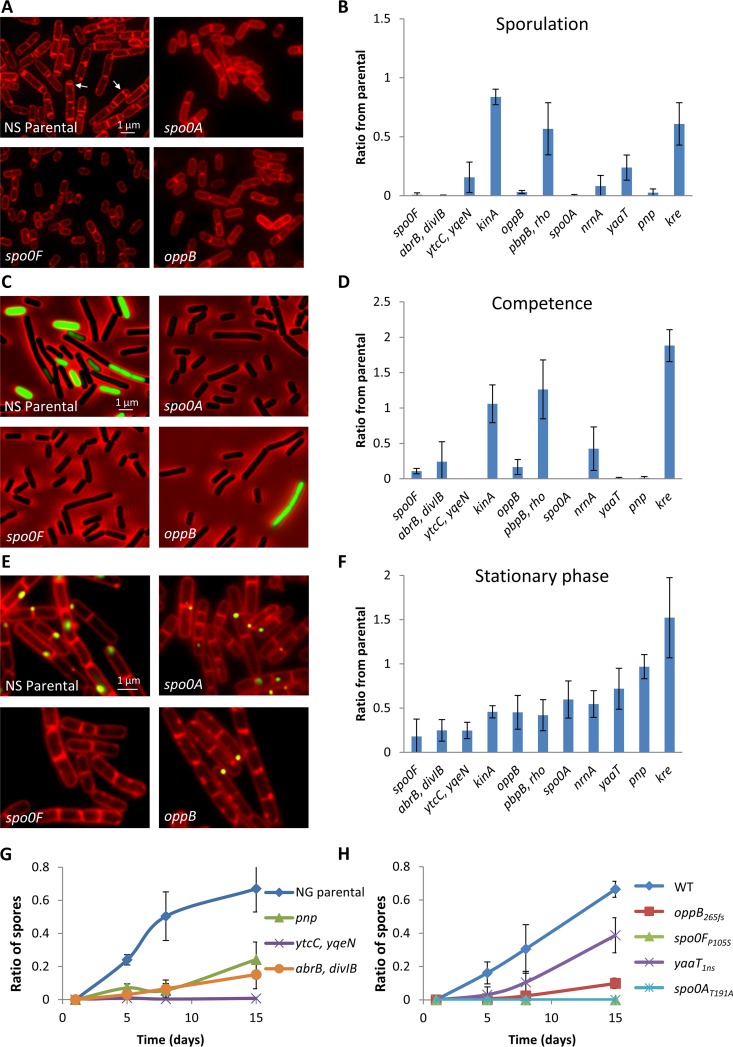
Morphomutants are defective in their ability to initiate sporulation, competence, and the stationary phase. (A and B) NS (RU9), NG (RU124), and morphomutant strains were induced to sporulate and polar septum formation was assessed at 2.5 h for the NS background or at 1.5 h for the NG background. (A) Signal from FM 4-64 membrane dye of three representative morphomutants and their corresponding NS (RU9) parental strain, with arrows indicating polar septum formation. (B) Quantification of polar septa formation in various morphomutants scored and normalized to the corresponding parental strain. The results are average values and the SD of three independent experiments. (C and D) NS (RU142), NG (RU156), and morphomutant strains harboring P*_comK_-gfp* were incubated in competence-inducing medium, and GFP-expressing cells were monitored at 3 h. (C) Overlay images of GFP (green) and phase-contrast (red) of three representative morphomutants and their corresponding NS (RU142) parental strain. (D) Quantification of GFP-expressing cells in the population in various morphomutants. Results were normalized to the corresponding parental strain and are presented as average values and the SD from three independent experiments. (E and F) NS (RU163), NG (RU174), and morphomutant strains harboring a *ylbP-gfp* fusion were grown to an OD_600_ of ∼1.5, and YlbP-GFP induction was detected. (E) Overlay images of YlbP-GFP (green) and FM 4-64 membrane dye (red) of three representative morphomutants and their corresponding NS parental strain (RU163). (F) YlbP-GFP-expressing cells were scored, and their ratios in the population were calculated. The results were normalized to the corresponding parental strain and are presented as average values and the SD from three independent experiments. (G and H) Colonies of the indicated strains were incubated for 15 days, and the cells were collected and visualized by light microscopy on various days. The cells and spores were counted, and their ratios were calculated for the different ages. (G) NG (RU124) parental strain and its derived indicated morphomutants. (H) WT (PY79) parental strain and its mutant derivatives obtained by site-directed mutagenesis. The results are presented as average values and the SD of three independent experiments.

The occurrence of mutations in the Spo0A pathway led to the hypothesis that the evolved morphomutants could be deficient in differentiation, an attribute that may facilitate their division under nutrient restrictive conditions. To investigate this possibility, we examined the ability of the morphomutant strains to differentiate into three major developmental fates: (i) sporulation, by following the formation of polar septa, the hallmark of sporulation initiation (27); (ii) competence, by employing a reporter harboring the promoter of the main competence transcription factor *comK* fused to *gfp* (P*_comK-_gfp*) (41); and (iii) stationary phase, by monitoring the expression of YlbP-GFP fusion, shown to be elevated during stationary phase (42) (Fig. S4A and B) and, accordingly, in cells of aging colonies (Fig. S4C). An apparent delay in polar septa formation was prominent in 10 of the 11 examined strains upon sporulation induction, with some displaying an almost complete sporulation halt (Fig. 3A and B and Fig. S5). Consistently, aging morphomutant colonies derived from a sporulating parental strain, which was capable of forming mature spores, exhibited a strong deficiency in spore formation kinetics within the colony, showing very few spores, if any, after 15 days of incubation (Fig. 3G and H and Fig. S6 and S7). In almost all sporulation-deficient mutants (8/10), a substantial defect in the induction of competence was observed (Fig. 3C and D). In line with these results, deficiency in entry to stationary phase was prominent in at least 7 of the 11 examined morphomutants (Fig. 3E and F). Taken together, the acquisition of mutations perturbing the Spo0A pathway yielded a group of morphomutants harboring differentiation deficiencies, with the majority (10/11) displaying measurable defects in sporulation, competence or stationary phase destinies. These results reflect the existence of a general strategy to tolerate colony aging by the acquisition of mutations that reduce the efficiency to differentiate.

10.1128/mBio.01414-19.4 FIG S4 Analysis of the YlbP-GFP expression pattern. (A) NS parental strain harboring *ylbP-gfp* fusion (RU163) was grown in LB and the culture was followed by determining the OD_600_ and using light microscopy. Cells expressing YlbP-GFP foci were counted, and their ratio in the population was calculated. The results are average values and SD of two independent experiments. (B) RU163 cells were grown as described in (A). Shown are overlay images of FM 4-64 membrane stain (red) and signal from YlbP-GFP (green) visualized at the indicated time points. (C) Five-day-old colonies of RU163 were visualized by light microscopy. Shown are overlay images of FM 4-64 membrane stain (red) and signal from YlbP-GFP (green) of cells visualized at the indicated days. Download FIG S4, TIF file, 2.7 MB. Copyright © 2019 Hashuel and Ben-Yehuda. 2019 Hashuel and Ben-Yehuda This content is distributed under the terms of the Creative Commons Attribution 4.0 International license.

10.1128/mBio.01414-19.5 FIG S5 Morphomutants are defective in their ability to enter sporulation. (A and B) NS (RU9), NG (RU124) and derived morphomutant strains were induced to sporulate and polar septum formation was assessed at 2.5 h (NS background) or 1.5 h (NG background). Signal from FM 4-64 membrane dye of NS-derived morphomutants (A) and NG-derived morphomutants (B) is shown. Arrows indicate polar septum formation. The quantified results are presented in Fig. 3B. Download FIG S5, TIF file, 2.7 MB. Copyright © 2019 Hashuel and Ben-Yehuda. 2019 Hashuel and Ben-Yehuda This content is distributed under the terms of the Creative Commons Attribution 4.0 International license.

10.1128/mBio.01414-19.6 FIG S6 NG morphomutants produce small amounts of spores during colony aging. Colonies of NG (RU124) parental strain and its derived morphomutants were incubated for 15 days. Samples were collected at various time points and visualized by light microscopy. Shown are representative phase-contrast images of the cells during colony aging. Arrows highlight spores. The quantified results are presented in Fig. 3G. Download FIG S6, TIF file, 2.7 MB. Copyright © 2019 Hashuel and Ben-Yehuda. 2019 Hashuel and Ben-Yehuda This content is distributed under the terms of the Creative Commons Attribution 4.0 International license.

10.1128/mBio.01414-19.7 FIG S7 Reconstructed morphomutants show a small number of spores during colony aging. Colonies of WT (PY79) parental strain and its mutant derivatives, obtained by site-directed mutagenesis, were incubated for 15 days. Samples were collected at the indicated time points and visualized by light microscopy. Shown are representative phase-contrast images of the cells during colony aging. Arrows highlight spores. The quantified results are presented in Fig. 3H. Download FIG S7, TIF file, 2.7 MB. Copyright © 2019 Hashuel and Ben-Yehuda. 2019 Hashuel and Ben-Yehuda This content is distributed under the terms of the Creative Commons Attribution 4.0 International license.

### Morphomutants mutated in genes involved in basic cellular processes are deficient in differentiation.

**FIG 4:**
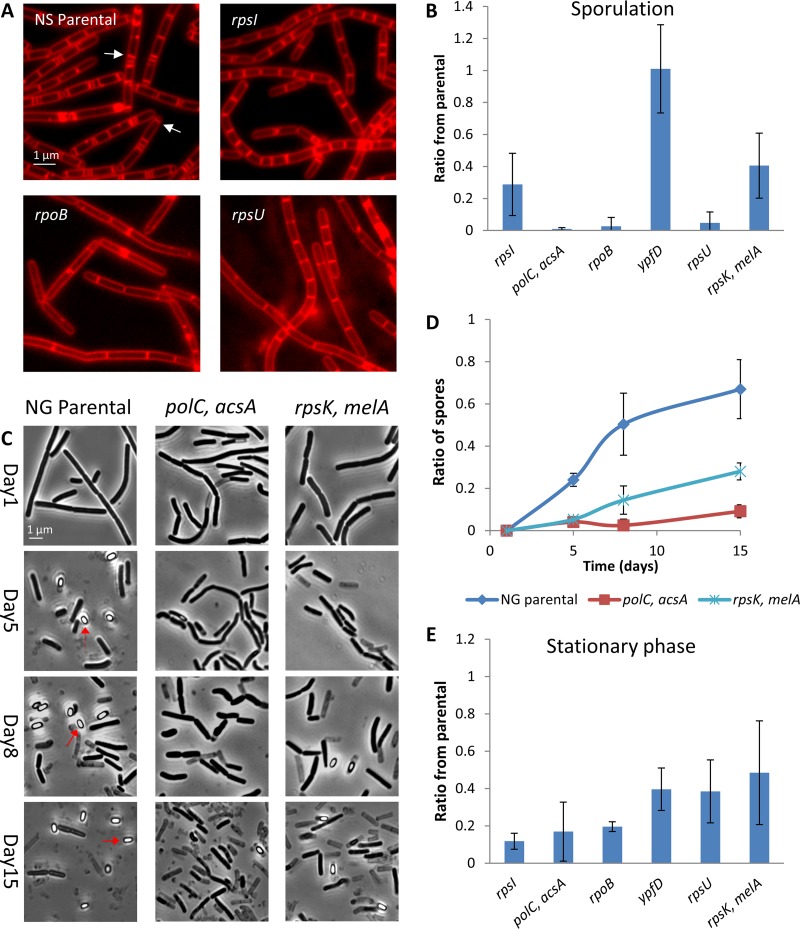
Morphomutants mutated in genes involved in basic cellular processes display nondifferentiation phenotypes. (A and B) NS (RU9), NG (RU124) and morphomutant strains, mutated in genes involved in general cellular processes, were induced to sporulate, and polar septa formation was assessed at 2.5 h for the NS background or at 1.5 h for the NG background. (A) Red FM 4-64 membrane dye of three representative morphomutants and their NS parental (RU9) strain, with arrows indicating polar septum formation. (B) The polar septum formation in various morphomutants was scored and normalized to the corresponding parental strain. The results are average values and the SD from three independent experiments. (C and D) Colonies of NG (RU124) parental strain and its derived morphomutants (*polC*, *acsA*, and *rpsK*, *melA*) were incubated for 15 days. Samples were collected at the indicated time points and visualized by light microscopy. (C) Representative phase-contrast images of cells during colony aging. Arrows highlight spores. (D) Cells and spores were counted, and their ratios were calculated at the indicated ages. The results are presented as average values and the SD from three independent experiments. (E) NS (RU163), NG (RU174), and morphomutant strains harboring a *ylbP-gfp* fusion were grown to an OD_600_ of ∼1.5, and YlbP-GFP induction was detected. YlbP-GFP-expressing cells were scored, and their ratios in the population were calculated. The results were normalized to the corresponding parental strain and are presented as average values and the SD from three independent experiments.

Apart from mutants in the Spo0A pathway, the morphomutants harbored mutations in genes related to basic cellular functions (Fig. 2A and Table S1B). Interestingly, several genes involved in elementary cellular processes were found to impact bacterial differentiation (see, for example, references 43 and 44). We therefore hypothesized that this group of morphomutants could be deficient in differentiation, similarly to those with mutations in the Spo0A pathway. Testing the ability of these strains to sporulate revealed that five of six exhibited a marked delay in initiating sporulation (Fig. 4A and B and Fig. S8). Moreover, morphomutant colonies derived from a sporulating strain showed a strong deficiency in spore formation during aging, with very few spores observed after 15 days of incubation (Fig. 4C and D). Lastly, although no significant effect on the transition to competence was detected in these mutants (data not shown), they displayed slower kinetics of entry into stationary phase than the parental strain (Fig. 4E). These results support the view that a plethora of mutations that develop during aging lead to the same outcome of evading differentiation.

10.1128/mBio.01414-19.8 FIG S8 Morphomutants mutated in genes involved in basic cellular processes show a delay in entry to sporulation. (A and B) NS (RU9), NG (RU124), and morphomutant strains, mutated in genes involved in general cellular processes, were induced to sporulate and polar septa formation was assessed at 2.5 h for NS background or 1.5 h for NG background. Shown is a signal from FM 4-64 membrane dye of NS-derived morphomutants and their parental strain (RU9) (A) and NG-derived morphomutants and their parental strain (RU124) (B). Arrows highlight polar septum formation. The quantified results are presented in Fig. 4A. Download FIG S8, TIF file, 2.9 MB. Copyright © 2019 Hashuel and Ben-Yehuda. 2019 Hashuel and Ben-Yehuda This content is distributed under the terms of the Creative Commons Attribution 4.0 International license.

### Advantageous features of morphomutant aging colonies.

**FIG 5:**
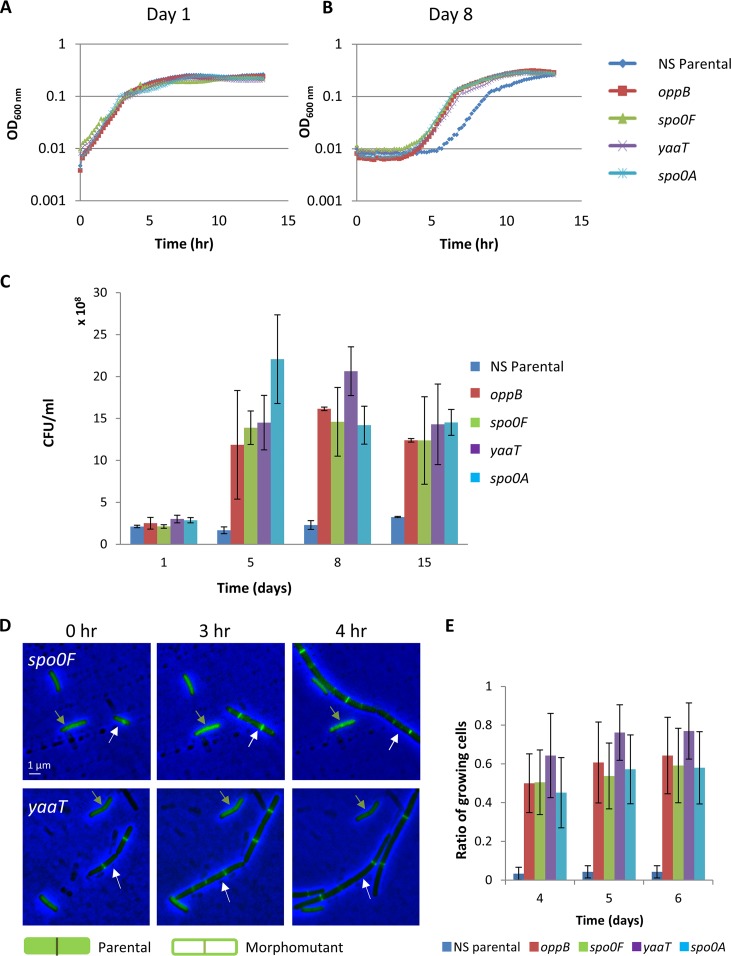
Morphomutants show beneficial properties during aging. (A and B) Colonies of NS (RU9) parental strain and four representative morphomutants were incubated for 8 days. Cells were collected at days 1 and 8 and then resuspended in fresh medium, and growth was monitored by determining the OD_600_. Growth curves of the strains at day 1 (A) and day 8 (B) are shown. The results are representative values of six independent experiments. (C) Single colonies of NS (RU9) and the listed representative morphomutants were incubated for 15 days. On the indicated days, single colonies were harvested, and cells were collected and plated for CFU. The results are presented as average values and the SD from three independent experiments. (D and E) Eight-day-old colonies of NS (RU23) strain, harboring both *ftsZ-gfp* and P*_rrnE_-gfp* (diffused pattern) fusions, and representative morphomutants harboring solely the *ftsZ-gfp* fusion were collected, mixed at a 1:1 ratio, and replated on the same LB agar pad. The cells were monitored using time-lapse microscopy. Of note, mCherry and CFP caused growth perturbation after aging and thus could not be utilized to differentiate between the mixed strains; therefore, P*_rrnE_-gfp* was added to the parental strain. (D) Phase-contrast (blue) and GFP (green) overlay images of the NS (RU23) parental strain and the *spo0F* (upper images) and *yaaT* (lower images) morphomutants. The legend shows the predicted GFP pattern from the parental (diffused GFP) and morphomutants (FtsZ-GFP rings). (E) Growing cells were counted, and their ratios in the entire cell population were calculated. The results are presented as average values and the SD from three independent experiments.

To further explore the morphomutants’ advantage during aging, we compared parental and mutant survival over time. Four representative morphomutant strains (*oppB*, *spo0F*, *yaaT*, and *spo0A*) displaying growth kinetics similar to that of the parental strain (Fig. 5A) were used for this analysis. A sharp increase (∼9-fold) in CFU was detected for all tested morphomutants during aging compared to the parental strain (Fig. 5C), reinforcing the view that the morphomutants are capable of dividing efficiently under restrictive conditions. Next, we monitored growth resumption of cells originating from aging colonies of morphomutant strains. Morphomutant cells deriving from 8-day-old colonies and further displayed a shorter lag phase, rapidly resuming growth compared to parental cells of similar age (Fig. 5A and B and Fig. S9A). Notably, the extent of the lag phase was not significantly influenced by the initial cell number (Fig. S9B). To precisely pursue this phenotype at a single cell resolution, we conducted a series of time-lapse microscopy experiments, where cells of an 8-day-old morphomutant colony were mixed with differentially labeled parental cells of the same age, and their return to growth was monitored. Indeed, morphomutant cells showed an apparent earlier growth resumption compared to the equivalent parental cells (Fig. 5D and E). Thus, we conclude that the examined aging morphomutants gained the capacity to both increase survival and shorten their lag phase when reinitiating growth.

10.1128/mBio.01414-19.9 FIG S9 The extent of the lag phase is not significantly affected by variation in the initial cell inoculum. (A) Eight-day-old colonies of the indicated NS parental (RU9) and 4 representative corresponding mutant strains were suspended to the same OD_600_ and replated for initial cell number evaluation using CFU analysis. (B) Colonies of NS (RU9) parental strain were diluted to different concentrations, measured by OD_600_ (×1, ×2, and ×3), and growth was monitored by determining the OD_600_. Of note, the lag phase remained similar among the NS parental samples, showing that the small difference of the initial CFU/ml indicated in panel A is unlikely to affect the length of the lag phase. *Spo0A* morphomutant growth curve is presented for comparison, to designate the shorter morphomutant lag phase. The results are representative values of four independent experiments. Download FIG S9, TIF file, 0.4 MB. Copyright © 2019 Hashuel and Ben-Yehuda. 2019 Hashuel and Ben-Yehuda This content is distributed under the terms of the Creative Commons Attribution 4.0 International license.

## DISCUSSION

**FIG 6:**
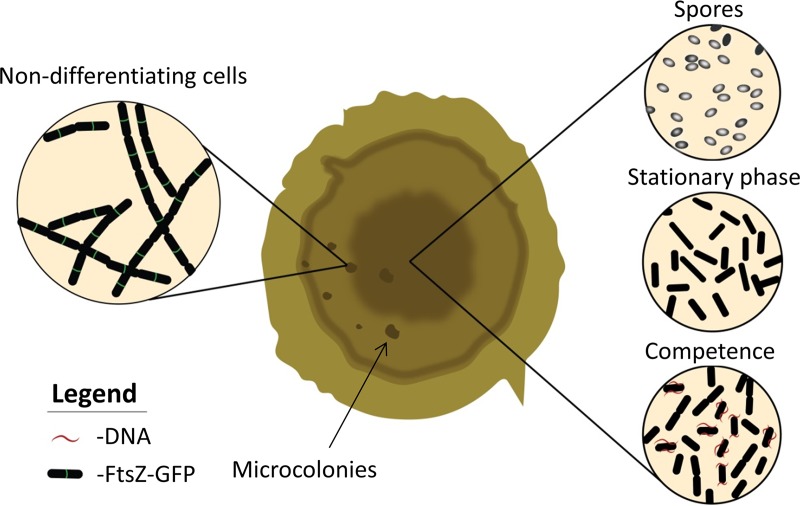
Model of the aging process of a B. subtilis colony. The colony is composed of different cell types, including spores, stationary-phase cells, and competent cells, all displaying a low growth rate. Over time, mutants in entering differentiation evolve and continue to divide under nutrient deprivation, giving rise to visible microcolonies.

Based on our findings, we propose that cells residing in aging colonies, coping with nutrient exhaustion, accumulate mutations in genes crucial for bacterial differentiation pathways, enabling their division under unfavorable conditions and the subsequent formation of microcolonies (Fig. 6). Thus, as an alternative to entering dormancy in order to avoid stress (see, for example, references 1, 15, 27, and 45), this trajectory allows bacteria to proliferate in challenging niches. By analogy to animal cells, these mutants resemble cancerous cells that accumulate mutations resulting in uncontrolled cell division and tumor formation (46). Temporarily, such bacterial mutants have a growth advantage, since they can rapidly resume division and flourish, whereas their neighboring cells are paused in a given differentiation state. Naturally, this rejuvenating phenotype would necessitate access to nutrients, which could be provided to the survivors by their perished siblings (7, 38, 47).

Although the leverage of overgrowing mutants is fairly intuitive in the immediate time scale, their long-term evolutionary advantage is ambiguous, since they possess fewer crucial features required for coping with stress, including spore formation, competence, and entering stationary phase. Furthermore, the advantage of population heterogeneity, i.e., cells exhibiting various fates, a concept standing at the base of evolution (see, for example, references 21, 48, and 49), is weakened in these nondifferentiating mutants. We therefore assume that such mutated strains might be unable to maintain species continuity in natural settings, and we hypothesize that nondifferentiating mutations repetitively arise in natural populations but revert or vanish in challenging niches. In agreement with this view, occasionally replating the morphomutants resulted in the appearance of repressors, reverting the altered shape phenotype of their parental morphomutant strain, thus pointing toward a potential drawback of the original mutation.

Only a few genes have been implicated in GASP phenotypes. Apart from *rpoS*, mutations accumulating during bacterial aging were assigned in E. coli to genes involved mainly in amino acid metabolism. This includes mutations in the leucine responsive regulatory gene *lrp*; in the gene *cstA*, which encodes an oligopeptide permease; and in the *ybeJ–gltJKL* operon, predicted to encode a high-affinity aspartate and glutamate ABC-type transporter (18). Similarly, Pseudomonas fluorescens Pf0-1 colonies were shown to generate cells that acquired mutations within the *rsmE* locus, regulating a pathway implicated mainly in metabolism and host colonization (35). In line with this view, prolonged incubation of B. subtilis cultures yielded adaptive isolates exhibiting alterations in the transcriptional profile of several metabolic pathways and suggesting that a key pathway in acquiring adaptive features is by harboring mutations in metabolic genes (8). The latter mutations were proposed to enable the cells to more efficiently rely on alternative sources of energy and biosynthetic substrates, including their siblings' remnants and internal stores (18). Here, we revealed an alternative pathway to overcome nutrient limitation by losing differentiation capabilities.

The notion that cells residing in long-term stationary phase are prone to accumulate mutations that contribute to strain durability is well established (see, for example, references 50 and 51). Diverse studies suggest various mechanisms to explain the source of the high mutation rate observed. Some studies support the view that this increase is caused by induced mechanisms in response to a specific selective pressure (see, for example, references 13 and 14), while others provide evidence that this elevation in adaptive mutations is solely due to advantageous features of the emerging mutants (see, for example, references 52 and 53). In this regard, we found that 5 to 20% of the progeny population of 25-day-old colonies displayed altered colony phenotypes. We further showed that all the sequenced morphomutants contained at least one mutation in their genome. It should be noted that we did not sequence the entire progeny population, and therefore we could not assess the precise mutant ratio. Nevertheless, it is reasonable to assume that the actual mutation rate could be even higher than the proportion of the morphomutant colonies in the population.

Interestingly, mutations in various genes, even in those that were not classically assigned to developmental pathways, led to the observed differentiation deficiency, indicating the existence of multiple paths to reach a similar consequence. The ease of obtaining such mutations suggests that they are frequent in nature, allowing bacterial growth under restrictive conditions. In line with this view, the prevalence of GASP mutations located within *rpoS*, which is involved in the transition to stationary phase in E. coli (18, 20), suggests that they could act similarly, reducing entry into the stationary state. This assumption, however, should be tested experimentally.

Thus far, bacterial cells facing nutrient deprivation have been shown to enter dormancy as a strategy to avoid stress. Numerous examples have been previously described, including sporulation and stationary phase, as well as persister cells, known to survive antibiotic treatments due to temporarily residing in a nondividing state (11, 15, 45). Here, we uncovered an opposing strategy in which mutant cells cope with a challenging niche by proliferating rather than stalling division. This strategy could be a major factor affecting the dynamics of bacterial populations in natural environments.

## MATERIALS AND METHODS

### Bacterial strains and plasmids.

The B. subtilis strains examined here were derivatives of the WT PY79 (54) and are listed in Table S1A and B; the primers are listed in Table S1C.

### General methods.

All general methods were carried out as described previously (55). Unless indicated otherwise, sporulation was induced by suspending cells in Schaeffer’s liquid medium (56), and heat kill analysis was carried out by incubating the cells for 30 min at 80°C. Additional molecular biology methods, including cloning, were carried out as described previously (57).

### Light microscopy.

To visualize cells and fluorescent signals, cells were placed on a microscope slide and covered with a poly-l-lysine-coated cover glass. To visualize FtsZ-GFP proteins, specimens were placed on thin 1×T-Base (55)–1% agarose pads. For time-lapse microscopy experiments (Fig. 5D and E), parental strains were differentially marked with P*_rrnE_-gfp* and placed over 1% agarose pads containing LB medium. Cells were visualized and photographed using an Axioobserver Z1 microscope (Zeiss) equipped with a CoolSnap HQII camera (Photometrics, Roper Scientific) or an Eclipse Ti (Nikon, Japan), equipped with a CoolSnap HQII camera. System control and image processing were performed using MetaMorph 7.7.5 software (Molecular Devices) or NIS Elements AR 4.3 (Nikon, Japan).

### Two-photon microscopy.

Colonies were lifted as a whole onto a slide, and colony regions were visualized and photographed using Ti Upright Nikon A1 MP multiphoton confocal microscope (Nikon) equipped with a GaAsP detectors 4 camera (Hamamatsu). Image processing was performed using NIS Elements AR 4.3.

### Colony imaging.

Colonies were observed and photographed using Discovery V20 stereoscope (Zeiss) equipped with Infinity1 camera (Luminera) or using Nikon D5100 camera equipped with Nikon 50-mm lens.

### Analyses of aging colonies. (i) Colony growth and aging.

For long-term colony aging experiments, bacterial strains (NS, NG, or WT and their derivatives) were diluted to 10^−4^, plated on LB agar (1.6%), and incubated at 30°C. At the required age, the colonies were further analyzed as described below, with NG- or WT-derived strains grown at 37°C, and the NS temperature-sensitive background strains grown at 30°C. The temperature sensitivity of RU9 derives from harboring the FtsZ-GFP fusion (58, 59). RU9 grown under the permissive temperature (30°C) does not exhibit a measurable phenotype. In all of the experiments, we present the mutant strain data compared to their corresponding parental strain.

**(ii) Imaging cells in aging colonies.** For cell imaging, colonies at the indicated ages were suspended in 500 μl of 1× phosphate-buffered saline (PBS) and visualized by light microscopy.

**(iii) Return to growth experiments in aging colonies.** At the indicated days, aging colonies were suspended in 500 μl of LB medium, and cultures were inoculated at an optical density at 600 nm (OD_600_) of 0.05, and the OD_600_ was measured over time in short intervals using a Spark 10M multimode microplate reader (Tecan) (Fig. 5A and B).

**(iv) Evaluating morphomutant frequencies in aging colonies.** Colonies at the indicated age were suspended in 100 μl LB diluted to 10^−3^ to 10^−5^ and replated on fresh LB agar plates (Fig. S3B and C). Progeny colonies were counted, and the ratio of altered-shape colonies was determined. To evaluate the altered-shape frequency within the microcolony-enriched regions at the indicated days, the colonies were divided into separate regions based on the presence of visible microcolonies. The cells were collected manually from microcolony-enriched regions (micro^+^) and from regions lacking visible microcolonies (micro^−^). Each region was suspended and plated separately on LB agar plates, and the ratio of the altered-shape colonies was determined. Statistical analyses and graphs were performed using Prism 7 (GraphPad). Significance was evaluated with a two-tail Mann-Whitney test (*P* = 0.00145) to determine the difference between micro^+^ and micro^–^ colonies.

**(v) Evaluation of cell viability in aging colonies.** Cells from an overnight culture were suspended in LB medium at an OD_600_ of 0.3, and 10 μl portions were spotted separately onto a single plate (in quadruplet) to enable the formation of a single colony on each plate (Fig. 5C). At days 1, 5, 8, and 15, the entire single colony was harvested, suspended in 3 ml of 1× PBS, disrupted two times using FastPrep (MP Biomedicals; 6.0 m/s, 40 s), diluted in 1× PBS to 10^−5^, and plated on LB plates at 30°C, and the CFU/ml was determined.

### Following entry to sporulation.

Cells were inoculated in casein hydrolysate medium (CH) (55) at an OD_600_ of 0.1 from an overnight CH culture. At an OD_600_ of 0.5 to 0.7, the cells were suspended in sporulation resuspension medium (55), followed by incubation at 30°C for NS background strains or at 37°C for NG and WT background strains. The cells were stained with 5 μg/ml FM 4-64 membrane dye (ThermoFisher Scientific) and visualized by using light microscopy every 30 min. The cells were counted, and the ratio of cells exhibiting a polar septum was determined.

### Defining the cell competence state.

Cells harboring P*_comK_-gfp* were inoculated at an OD_600_ of 0.1 in MC×1 minimal medium (80 mM K_2_HPO_4_, 30 mM KH_2_PO_4_, 2% glucose, 30 mM trisodium citrate, 22 μg/ml ferric ammonium citrate, 0.1% casein hydrolysate, 0.2% potassium glutamate) for competence induction. Cells were visualized by light microscopy 3 h after inoculation. The cells were counted, and the ratio of GFP- expressing cells was determined.

### Quantifying entry into stationary phase.

Strains harboring *ylbP-gfp* fusion were grown in 3 ml of LB medium at 23°C for ∼15 h, and the cells were visualized using light microscopy. The cells were counted, and the ratio of bacteria that exhibited YlbP-GFP foci was determined.

### Whole-genome sequencing.

Genomic DNA was extracted from mutant and WT strains using a Wizard genomic DNA purification kit (Promega). Libraries were prepared by using a Nextra XT kit (Illumina) and sequenced by pair end sequencing in a MiSeq sequencer (Illumina), with a fragment size of 250 bp. Quality assessment was done with the software FastQC (v0.10.1). Sequence reads were aligned using NCBI B. subtilis PY79 genome (GenBank accession no. CP004405.1).

### Site-directed mutagenesis.

Construction of point mutant strains was carried out as described previously (60). Briefly, to replace the WT with the mutated allele found in a morphomutant genome, a PCR product containing ∼500 bp upstream and ∼500 bp downstream of the desired mutation was amplified using chromosomal DNA purified from PY79 as the template (Table S1A). The PCR product was digested with BamHI and SalI and cloned into the BamHI and SalI sites of pMINImad2. Site-directed mutagenesis was conducted on this plasmid to change the codon encoding the WT allele to the mutated allele using primers containing the desired mutation (Table S1C) and a QuikChange II kit (Stratagene) to create the desired plasmids. The plasmids were sequenced to ensure the presence of the mutation. Each mutated plasmid was then introduced into PY79 genome via a single-crossover integration by transformation at the restrictive temperature for plasmid replication (37°C), using *mls* resistance as a selective marker. To evict the plasmid, the strain was incubated in 3 ml of LB medium at a permissive temperature for plasmid replication (23°C) for 14 h, diluted 30-fold into fresh LB medium, and incubated at 23°C for another 8 h. The cells were then serially diluted and plated on LB agar at 37°C. Individual colonies were patched onto both LB plates and LB plates containing *mls* to identify *mls*-sensitive colonies that had evicted the plasmid. Chromosomal DNA from colonies that had excised the plasmid was purified and screened by PCR to determine which isolate had retained the mutated allele. Sequence analysis was used to validate allele replacement.

### Plasmid construction.

pET770 (*ylbP-gfp-spc*) was constructed by amplifying the C terminus of *ylbP* from gDNA of the B. subtilis strain (PY79) with the primers ylbP-U-EcoRI and ylbP-L-XhoI (Table S1C). The PCR-amplified DNA was digested with EcoRI and XhoI and was cloned into pKL147 (61) digested with the same enzymes.

### Estimation of ratio variance.

The ratios were defined as μ_X_/μ_Y_, and the variance (*Var*) was estimated using the following formula:

$\mathrm{Var}\left(R\right)\approx \frac{1}{\mu_{x}^{2}\;}\;\left(r^{2\;}\sigma_{x}^{2}+\sigma_{y}^{2}-2r\sigma_{\mathrm{xy}}\right)$ (62).

The standard deviation (SD) was calculated to be the square root of the variance.
