# Supplementary material for: Aging of a Bacterial Colony Enforces the Evolvement of Nondifferentiating Mutants
Source: mBio. 2019 Sep 3;10(5):e01414-19. doi: 10.1128/mBio.01414-19 (PMC6722413; doi:10.1128/mBio.01414-19)
Supplement: TABLE S1 [file mBio.01414-19-st001.docx]

**Table S1. List of bacterial strains and primers used in this study**

**S1A. List of bacterial strains used in this study**

| **Strain Name** | **Genotype** | **Description** | **Phenotype** |
| --- | --- | --- | --- |
| PY79 | *B. subtilis* | Wild type ([1](#_ENREF_1)) |  |
| SB177 | *ftsZ-gfp-kan* (temperature sensitive-ts) | Lab stock | FtsZ-GFP ts, Kan^R^ |
| RL2087 | *spoIIE-mls* (*spoIIE* is intact) | Lab stock | Mls^R^ |
| RL76 | *spoIIIE36* | Lab stock ([2](#_ENREF_2)) | Spo^-^ |
| AR16 | *amyE::*P*_rrnE_-gfp-spc* | Lab stock ([3](#_ENREF_3)) | GFP^+^, Spc^R^ |
| SB306 | *amyE::*P*_racA_-gfp-racA-cat* | Lab stock | GFP^+^ , Cm^R^ |
| YA99 | P*_comK_-gfp-spc* | Lab stock | GFP^+^ , Spc^R^ |
| RL1964 | s*poIIE::kan* | Lab stock ([4](#_ENREF_4)) | Spo^-^, Kan^R^ |
| ET770 | *ylbP-gfp-spc* | Lab stock | YlbP-GFP, Spc^R^ |
| RU9 | *spoIIIE36, spoIIE-mls, ftsZ-gfp-kan* (ts) | Constructed by transforming gDNA from strain SB177 into RU11 strain | Spo^-^, FtsZ-GFP ts, Mls^R^, Kan^R^ |
| RU11 | *spoIIIE36, spoIIE-mls* | Constructed by transforming gDNA from strain RL2087 into RL76 strain | Spo^-^, Mls^R^ |
| RU23 | *spoIIIE36, spoIIE-mls, ftsZ-gfp-kan, amyE::*P*_rrnE_-gfp-spc* | Constructed by transforming gDNA from strain AR16 into RU9 strain | Spo^-^, FtsZ-GFP ts, GFP^+^(cytoplasmic), Mls^R^, Kan^R^, Spc^R^ |
| RU124 | *gerAA::cm, gerBA::spc, gerD::kan, gerKA::mls* | Constructed using Gibson assembly kit (NEB, USA) utilizing primers: *gerA*-P1-P4, *gerB*-P1-P4, *gerD*-P1-P4, *gerKA*-P1-P4 (Table S1C) | Ger ^-^, Cm^R^, Spc^R^, Kan^R^, Mls^R^ |
| RU125 | *spoIIIE36, spoIIE-mls, amyE::*P*_racA_-gfp-racA-cat* | Constructed by transforming gDNA from strain SB306 into RU11 strain | Spo^‑^, GFP^+^ , Mls^R^, Cm^R^ |
| RU101 | *oppB_265fs_* | Site directed mutagenesis to PY79, using primers: *oppB-ins_2nt_after_795-antisense oppB-ins_2nt_after_795* (Table S1C) | Altered shape colony, deficient in sporulation, competence and stationary phase (this study) |
| RU104 | *spo0F_P105S_* | Site directed mutagenesis to PY79, using primers: *spo0F-c313t_antisense spo0F-c313t* (Table S1C) | Altered shape colony, deficient in sporulation, competence and stationary phase (this study) |
| RU105 | *yaaT_1ns_* | Site directed mutagenesis to PY79, using primers: *yaaT-t202a_antisense*  *yaaT-t202a* (Table S1C) | Altered shape colony, deficient in sporulation and competence (this study) |
| RU106 | *spo0A_T191A_* | Site directed mutagenesis to PY79, using primers: *spo0A-a580g_antisense spo0A-a580g* (Table S1C) | Altered shape colony, deficient in sporulation, competence and stationary phase (this study) |
| RU139 | *oppB_265fs_ , spoIIE::kan,* P*_comK_-gfp-spc* | Constructed by sequentially transforming gDNA from strain YA99 and RL1964 into RU101 | Spo^-^, GFP^+^, kan^R^, spc^R^ , Altered shape colony, deficient in sporulation, competence and stationary phase (this study) |
| RU140 | *spo0A_T191A_* , s*poIIE::kan,* P*_comK_-gfp-spc* | Constructed by sequentially transforming gDNA from strain YA99 and RL1964 into RU106 | Spo^-^, GFP^+^, Kan^R^, Spc^R^ , Altered shape colony, deficient in sporulation, competence and stationary phase (this study) |
| RU142 | *spoIIE::kan,* P*_comK_-gfp-spc* | Constructed by transforming gDNA from strain YA99 into RL1964 | Spo^-^, GFP^+^, Kan^R^, Spc^R^ |
| RU143 | *spo0F_P105S_ ,spoIIE::kan,* P*_comK_-gfp-spc* | Constructed by sequentially transforming gDNA from strain YA99 and RL1964 into RU104 | Spo^-^, GFP^+^, Kan^R^, Spc^R^ , Altered shape colony, deficient in sporulation, competence and stationary phase (this study) |
| RU144 | *yaaT_1ns_*, *spoIIE::Kan,* P*_comK_-gfp-spc* | Constructed by sequentially transforming gDNA from strain YA99 and RL1964 into RU105 | Spo^-^, GFP^+^, Kan^R^, Spc^R^ , Altered shape colony, deficient in sporulation and competence (this study) |
| RU145 | *ypfD_H30P_, spoIIIE36, spoIIE-mls, ftsZ-gfp-kan,* P*_comK_-gfp-spc* | Constructed by transforming gDNA from strain YA99 into AG13 | Spo^-^, FtsZ-GFP ts, GFP^+^ (cytoplasmic), Mls^R^, Kan^R^, Spc^R^ ,  Altered shape colony, deficient in stationary phase (this study) |
| RU146 | *rpsU_KRKF54RVDL_ ,spoIIIE36, spoIIE-mls, ftsZ-gfp-kan*, P*_comK_-gfp-spc* | Constructed by transforming gDNA from strain YA99 into AG14 | Spo^-^, FtsZ-GFP ts, GFP^+^(cytoplasmic), Mls^R^, Kan^R^, Spc^R^ , Altered shape colony, deficient in sporulation and stationary phase (this study) |
| RU147 | *rpsI_105fs,_ spoIIIE36, spoIIE-mls, ftsZ-gfp-kan*, P*_comK_-gfp-spc* | Constructed by transforming gDNA from strain YA99 into AG15 | Spo^-^, FtsZ-GFP ts, GFP^+^(cytoplasmic), Mls^R^, Kan^R^, Spc^R^ , Altered shape colony, deficient in sporulation and stationary phase (this study) |
| RU148 | *rpoB_P520L_*,*spoIIIE36, spoIIE-mls, ftsZ-gfp-kan*, P*_comK_-gfp-spc* | Constructed by transforming gDNA from strain YA99 into AG12 | Spo^-^, FtsZ-GFP ts, GFP^+^(cytoplasmic), Mls^R^, Kan^R^, Spc^R^ , Altered shape colony, deficient in sporulation and stationary phase (this study) |
| RU149 | *gerBA::tet* | Constructed using Gibson assembly kit (NEB, USA) utilizing primers *gerB*-P1-P4 (Table S1C) | Ger^-^, Tet^R^ |
| RU156 | *gerAA::cm, gerBA::tet, gerD::kan, gerKA::mls,* P*_comK_-gfp-spc* | Constructed by sequentially transforming gDNA from strain YA99 and RU149 into RU124 | Ger^-^, GFP^+^, Cm^R^, Tet^R^, Kan^R^, Mls^R^, Spc^R^ |
| RU158 | *polC_E678D_, acsA_165fs_, gerAA::cm, gerBA::tet, gerD::kan, gerKA::mls*, P*_comK_-gfp-spc* | Constructed by sequentially transforming gDNA from strain YA99 and RU149 into AG16 | Ger^-^, GFP^+^, Cm^R^, Tet^R^, Kan^R^, Mls^R^, Spc^R^ , Altered shape colony, deficient in sporulation and stationary phase (this study) |
| RU159 | *pnp_M1I_, gerAA::cm, gerBA::tet, gerD::kan, gerKA::mls*, P*_comK_-gfp-spc* | Constructed by sequentially transforming gDNA from strain YA99 and RU149 into AG9 | Ger^-^, GFP^+^, Cm^R^, Tet^R^, Kan^R^, Mls^R^, Spc^R^ , Altered shape colony, deficient in sporulation and competence (this study) |
| RU160 | *ytcC_381fs_, yqeN_E330K_, cypC_Y59H,_ yprB_W173L_, gerAA::cm, gerBA::tet, gerD::kan, gerKA::mls*, P*_comK_-gfp-spc* | Constructed by sequentially transforming gDNA from strain YA99 and RU149 into AG11 | Ger^-^, GFP^+^, Cm^R^, Tet^R^, Kan^R^, Mls^R^, Spc^R^ , Altered shape colony, deficient in sporulation, competence and stationary phase (this study) |
| RU161 | *rpsK_R124C_, melA_Q84R_, gerAA::cm, gerBA::tet, gerD::kan, gerKA::mls*, P*_comK_-gfp-spc* | Constructed by sequentially transforming gDNA from strain YA99 and RU149 into AG17 | Ger^-^, GFP^+^, Cm^R^, Tet^R^, Kan^R^, Mls^R^, Spc^R^ , Altered shape colony, deficient in sporulation (this study) |
| R162 | *abrB_M54ns_,divIB_105fs_, gerAA::cm, gerBA::tet, gerD::kan, gerKA::mls*, P_comK_*-gfp-spc* | Constructed by sequentially transforming gDNA from strain YA99 and RU149 into AG10 | Ger^-^, GFP^+^, Cm^R^, Tet^R^, Kan^R^, Mls^R^, Spc^R^ , Altered shape colony, deficient in sporulation, competence and stationary phase (this study) |
| RU163 | *spoIIE::kan, ylbP-gfp-spc* | Constructed by transforming gDNA from strain ET770 into RL1964 | Spo^-^, YlbP-GFP, Kan^R^, Spc^R^ |
| RU164 | *oppB_265fs_,* *spoIIE::kan, ylbP-gfp-spc* | Constructed by sequentially transforming gDNA from strain RL1964 and ET770 into RU101 | Spo^-^, YlbP-GFP, Kan^R^, Spc^R^ , Altered shape colony, deficient in sporulation, competence and stationary phase (this study) |
| RU165 | *spo0F_P105S_, spoIIE::kan, ylbP-gfp-spc* | Constructed by sequentially transforming gDNA from strain RL1964 and ET770 into RU104 | Spo^-^, YlbP-GFP, Kan^R^, Spc^R^ , Altered shape colony, deficient in sporulation, competence and stationary phase (this study) |
| RU167 | *yaaT_1ns_, spoIIE::kan, ylbP-gfp-spc* | Constructed by sequentially transforming gDNA from strain RL1964 and ET770 into RU105 | Spo^-^, YlbP-GFP, Kan^R^, Spc^R^ , Altered shape colony, deficient in sporulation and competence (this study). |
| RU168 | *kre_41fs_, spoIIIE36, spoIIE-mls, ftsZ-gfp-kan*, *ylbP-gfp-spc* | Constructed by transforming gDNA from strain ET770 into AG6 | Spo^-^, FtsZ-GFP ts, YlbP-GFP, Mls^R^, Kan^R^, Spc^R^ , Altered shape colony, deficient in sporulation (this study) |
| RU169 | *nrnA_148fs_, spoIIIE36, spoIIE-mls, ftsZ-gfp-kan, ylbP-gfp-spc* | Constructed by transforming gDNA from strain ET77 into AG7 | Spo^-^, FtsZ-GFP ts, YlbP-GFP, Mls^R^, Kan^R^, Spc^R^ , Altered shape colony, deficient in sporulation and competence (this study) |
| RU170 | *pbpB_G66C,_ rho_G446E_, spoIIIE36, spoIIE-mls, ftsZ-gfp-kan, ylbP-gfp-spc* | Constructed by transforming gDNA from strain ET770 into AG8 | Spo^-^, FtsZ-GFP ts, YlbP-GFP, Mls^R^, Kan^R^, Spc^R^ , Altered shape colony, deficient in sporulation and stationary phase (this study) |
| RU171 | *pnp_M1I_, gerAA::cm, gerBA::tet, gerD::kan, gerKA::mls*, *ylbP-gfp-spc* | Constructed by sequentially transforming gDNA from strain ET770 and RU149 into AG9 | Ger^-^, YlbP-GFP, Cm^R^, Tet^R^, Kan^R^, Mls^R^, Spc^R^ , Altered shape colony, deficient in sporulation and competence (this study) |
| RU172 | *ytcC_381fs_,yqeN_E330K_, cypC_Y59H,_  yprB_W173L_, gerAA::cm, gerBA::tet, gerD::kan, gerKA::mls*, *ylbP-gfp-spc* | Constructed by sequentially transforming gDNA from strain ET770 and RU149 into AG11 | Ger^-^, YlbP-GFP, Cm^R^, Tet^R^, Kan^R^, Mls^R^, Spc^R^ , Altered shape colony, deficient in sporulation, competence and stationary phase (this study) |
| RU173 | *abrB_M54ns_, divIB_105fs_, gerAA::cm, gerBA::tet, gerD::kan, gerKA::mls, ylbP-gfp-spc* | Constructed by sequentially transforming gDNA from strain ET770 and RU149 into AG10 | Ger^-^, YlbP-GFP, Cm^R^, Tet^R^, Kan^R^, Mls^R^, Spc^R^ , Altered shape colony, deficient in sporulation, competence and stationary phase (this study) |
| RU174 | *gerAA::cm, gerBA::tet, gerD::kan, gerKA::mls*, *ylbP-gfp-spc* | Constructed by sequentially transforming gDNA from strain ET770 and RU149 into R124 | Ger^-^, YlbP-GFP, Cm^R^, Tet^R^, Kan^R^, Mls^R^, Spc^R^ |
| RU175 | *kre_41fs_, spoIIIE36, spoIIE-mls, ftsZ-gfp-kan*, P*_comK_-gfp-spc* | Constructed by transforming gDNA from strain YA99 into AG6 | Spo^-^, FtsZ-GFP ts, GFP^+^(cytoplasmic), Mls^R^, Kan^R^, Spc^R^ , Altered shape colony, deficient in sporulation (this study) |
| RU176 | *pbpB_G66C,_ rho_G446E_, spoIIIE36, spoIIE-mls, ftsZ-gfp-kan*, P*_comK_-gfp-spc* | Constructed by transforming gDNA from strain YA99 into AG8 | Spo^-^, FtsZ-GFP ts, GFP^+^(cytoplasmic), Mls^R^, Kan^R^, Spc^R^ , Altered shape colony, deficient in sporulation and stationary phase (this study) |
| RU177 | *nrnA_148fs_, spoIIIE36, spoIIE-mls, ftsZ-gfp-kan*, P*_comK_-gfp-spc* | Constructed by transforming gDNA from strain YA99 into AG7 | Spo^-^, FtsZ-GFP ts, GFP^+^(cytoplasmic), Mls^R^, Kan^R^, Spc^R^ , Altered shape colony, deficient in sporulation and competence (this study) |
| RU194 | *kinA_418fs_, spoIIIE36, spoIIE-mls, ftsZ-gfp-kan*, P*_comK_-gfp-spc* | Constructed by transforming gDNA from strain YA99 into AG5 | Spo^-^, FtsZ-GFP ts, GFP^+^(cytoplasmic), Mls^R^, Kan^R^, Spc^R^ , Altered shape colony, deficient in stationary phase (this study) |
| RU195 | *kinA_418fs_, spoIIIE36, spoIIE-mls, ftsZ-gfp-kan*, *ylbP-gfp-spc* | Constructed by transforming gDNA from strain ET770 into AG5 | Spo^-^, FtsZ-GFP ts, YlbP-GFP, Mls^R^, Kan^R^, Spc^R^ , Altered shape colony, deficient in stationary phase (this study) |
| RU196 | *ypfD_H30P_, spoIIIE36, spoIIE-mls, ∆ftsZ-gfp-kan (lost)*, *ylbP-gfp-spc* | Constructed by transforming gDNA from strain ET770 into AG13 | Spo^-^, YlbP-GFP, Mls^R^, Kan^R^, Spc^R^ , Altered shape colony, deficient in stationary phase (this study) |
| RU197 | *rpoB_P520L_, spoIIIE36, spoIIE-mls, ftsZ-gfp-kan*, *ylbP-gfp-spc* | Constructed by transforming gDNA from strain ET770 into AG12 | Spo^-^, FtsZ-GFP ts, YlbP-GFP, Mls^R^, Kan^R^, Spc^R^ , Altered shape colony, deficient in sporulation and stationary phase (this study) |
| RU198 | *rpsU_KRKF54RVDL_, spoIIIE36, spoIIE-mls, ftsZ-gfp-kan*, *ylbP-gfp-spc* | Constructed by transforming gDNA from strain ET770 into AG14 | Spo^-^, FtsZ-GFP ts, YlbP-GFP, Mls^R^, Kan^R^, Spc^R^ , Altered shape colony, deficient in sporulation and stationary phase (this study) |
| RU199 | *rpsI_105fs_, spoIIIE36, spoIIE-mls, ftsZ-gfp-kan*, *ylbP-gfp-spc* | Constructed by transforming gDNA from strain ET770 into AG15 | Spo^-^, FtsZ-GFP ts, YlbP-GFP, Mls^R^, Kan^R^, Spc^R^ , Altered shape colony, deficient in sporulation and stationary phase (this study) |
| RU200 | *rpsK_R124C,_ melA_Q84R_, gerAA::cm, gerBA::tet, gerD::kan, gerKA::mls*, *ylbP-gfp-spc* | Constructed by sequentially transforming gDNA from strain ET770 and RU149 into AG17 | Ger^-^, YlbP-GFP, Mls^R^, Kan^R^, Spc^R^ , Altered shape colony, deficient in sporulation (this study) |
| RU201 | *spo0A_T191A_, ylbP-gfp-spc* | Constructed by *SPP1* mediated transduction of pET770 to RU106 | YlbP-GFP, Spc^R^ , Altered shape colony, deficient in sporulation, competence and stationary phase (this study) |
| RU202 | *polC_E678D,_ acsA_165fs_, gerAA::cm, gerBA::tet, gerD::kan, gerKA::mls*, *ylbP-gfp-spc* | Constructed by sequentially transforming gDNA from strain ET770 and RU149 into AG16 | Ger^-^, YlbP-GFP, Cm^R^, Tet^R^, Kan^R^, Mls^R^, Spc^R^ , Altered shape colony, deficient in sporulation and stationary phase (this study) |

*All strains originated from RU9 parental strain possess ts phenotype.

*GFP^+^ represents cytoplasmic GFP being expressed according to the gene noted in the genotype.

*Spo^-^ represents non-sporulating phenotype

*Ger^-^ represents non-germinating phenotype

**S1B. List of bacterial mutants discovered in this study**

| **Strain** | **Parental** | **Mutated Gene** | **Mutation** | **CDS position** | **Protein Length** | **Protein effect** | **Allele** |
| --- | --- | --- | --- | --- | --- | --- | --- |
| AG1 | RU9 | *oppB* | +AT | 795 | 311aa | Frameshift (I265-end) | *oppB_265fs_* |
| AG2 | RU9 | *spo0F* | G🡪A | 313 | 124aa | Substitution (P105S) | *spo0F_P105S_* |
| AG3 | RU9 | *yaaT* | T🡪A | 2 | 275aa | Nonsense (M1-Stop codon) | *yaaT_1ns_* |
| AG4 | RU9 | *spo0A* | T🡪C | 580 | 267aa | Substitution (T194A) | *spo0A_T191A_* |
| AG5 | RU9 | *kinA* | +T | 1254 | 606aa | Frameshift (L418- end) | *kinA_418fs_* |
| AG6 | RU9 | *kre* | +T | 121 | 154aa | Frameshift (K41-end) | *kre_41fs_* |
| AG7 | RU9 | *nrnA* | -C | 444 | 313aa | Frameshift (L148-end) | *nrnA_148fs_* |
| AG8 | RU9 | *pbpB* | G🡪T | 196 | 716aa | Substitution (G66C) | *pbpB_G66C_* |
|  |  | *rho* | C🡪T | 1007 | 427aa | Substitution (G336E) | *rho_G446E_* |
| AG9 | RU124 | *pnp* | G🡪T | 3 | 271aa | Substitution (M1I) | *pnp_M1I_* |
| AG10 | RU124 | *abrB* | G🡪T | 162 | 94aa | Nonsense (M54-stop codon) | *abrB_M54ns_* |
|  |  | *divIB* | -A | 315 | 263aa | Frameshift (K105-end) | *divIB_105fs_* |
| AG11 | RU124 | *ytcC* | -A | 1142 | 407aa | Frameshift (N381-end) | *ytcC_381fs_* |
|  |  | *yqeN* | C🡪T | 988 | 347aa | Substitution (E330K) | *yqeN_E330K_* |
|  |  | *cypC** | T🡪C | 175 | 417aa | Substitution (Y59H) | *cypC_Y59H_* |
|  |  | *yprB** | C🡪A | 518 | 413aa | Substitution (W173L) | *yprB_W173L_* |
| AG12 | RU9 | *rpoB* | C🡪T | 1559 | 1193aa | Substitution (P520L) | *rpoB_P520L_* |
| AG13 | RU9 | *ypfD (rpsA like)* | T🡪G | 89 | 382aa | Substitution (H30P) | *ypfD_H30P_* |
| AG14 | RU9 | *rpsU* | ∆C_160_-T_178_ | 160 | 57aa | Substitution (KRKF54RVDL) | *rpsU_KRKF54RVDL_* |
| AG15 | RU9 | *rpsI* | +A | 346 | 130aa | Frameshift (T105-end) | *rpsI_105fs_* |
| AG16 | RU124 | *polC* | A🡪C | 2034 | 1437aa | Substitution (E678D) | *polC_E678D_* |
|  |  | *acsA* | +G | 493 | 572aa | Frameshift (L165-end) | *acsA_165fs_* |
| AG17 | RU124 | *rpsK* | C🡪T | 370 | 131aa | Substitution (R124C) | *rpsK_R124C_* |
|  |  | *melA* | A🡪G | 251 | 432aa | Substitution (Q84R) | *melA_Q84R_* |

ns= nonsense mutation fs= frameshift mutation

*These two additional mutations were found in AG11 genome, but further analysis showed that these genes have no effect on sporulation or colony morphology.

**S1C. List of primers used in this study**

| **Primer name** | **Primer sequence** |
| --- | --- |
| *gerA*-P1 | 5’-ttcgaacggtccagcatgtgaa-3’ |
| *gerA*-P2 | 5’-ctgagcgagggagcagaa tatgaaagcggaggatacgaagtggc-3’ |
| *gerA*-P3 | 5’-gttgaccagtgctccctg caaatacaatgcttggggccggactt-3’ |
| *gerA*-P4 | 5’-cgtcatccggccagagagaaaaat-3’ |
| *gerB*-P1 | 5’-ctatatacccgtccacatggggaa-3’ |
| *gerB*-P2 | 5’-ctgagcgagggagcagaa ggaagcgttttctttgaagggg-3’ |
| *gerB*-P3 | 5’-gttgaccagtgctccctg atcagcagcacattgattggtgcc-3’ |
| *gerB*-P4 | 5’-aaaaacgtcgcagccagcattgcagt-3’ |
| *gerD*-P1 | 5’-gacaatgtgaccaagcaagtttccgg-3’ |
| *gerD*-P2 | 5’-ctgagcgagggagcagaa tcgctgttcgcgatttttgagg-3’ |
| *gerD*-P3 | 5’-gttgaccagtgctccctg taaagggaaagccgggatctggaatc-3’ |
| *gerD*-P4 | 5’-ccttcgatttgaagagctgcctgaag-3’ |
| *gerKA*-P1 | 5’-gcagtgctgtcagatgacatcgcatttc-3’ |
| *gerKA*-P2 | 5’-ctgagcgagggagcagaa agggttagatgcggtataacttctccgc-3’ |
| *gerKA*-P3 | 5’-gttgaccagtgctccctg tgcttttgagcgtcattgtgctgc-3’ |
| *gerKA*-P4 | 5’-ccttttcaagctctttgttgagcgc-3’ |
| *oppB-ins_2nt_after_795-antisense* | 5’-cgtataatcacggtttgtataatactgttgacgaagtgtgca-3’ |
| *oppB-ins_2nt_after_795* | 5’-tgcacacttcgtcaacagtattatacaaaccgtgattatacg-3’ |
| *spo0F-c313t_antisense* | 5’-tcgtcgatgtcaaacgacttggcaaagtgcgtc-3’ |
| *spo0F-c313t* | 5’-gacgcactttgccaagtcgtttgacatcgacga-3’ |
| *yaaT-t202a_antisense* | 5’-gacaccaattacattgtactagcttatccctcctgcaa-3’ |
| *yaaT-t202a* | 5’-ttgcaggagggataagctagtacaatgtaattggtgtc-3’ |
| *spo0A-a580g_antisense* | 5’-cggatagaggacttttgcaatgctgccgagcaatt-3’ |
| *spo0A-a580g* | 5’-aattgctcggcagcattgcaaaagtcctctatccg-3’ |
| *ylbP-U-EcoRI* | 5’- taggaattcgtctgcttatcaactataaaacgctgg-3’ |
| *ylbP-L-XhoI* | 5’- tagctcgagtgcagactctcccggtgt-3’ |

**References**

1. Youngman P, Perkins JB, Losick R. 1984. Construction of a cloning site near one end of Tn917 into which foreign DNA may be inserted without affecting transposition in *Bacillus subtilis* or expression of the transposon-borne *erm* gene. Plasmid 12:1-9.

2. Wu LJ, Errington J. 1994. *Bacillus subtilis* SpoIIIE protein required for DNA segregation during asymmetric cell division. Science 264:572-5.

3. Rosenberg A, Sinai L, Smith Y, Ben-Yehuda S. 2012. Dynamic expression of the translational machinery during *Bacillus subtilis* life cycle at a single cell level. PLoS One 7:e41921.

4. Arigoni F, Duncan L, Alper S, Losick R, Stragier P. 1996. SpoIIE governs the phosphorylation state of a protein regulating transcription factor sigma F during sporulation in *Bacillus subtilis*. Proc Natl Acad Sci U S A 93:3238-42.
